# Development of an Effective Acne Treatment Based on CBD and Herbal Extracts: Preliminary *In Vitro*, *Ex Vivo,* and Clinical Evaluation

**DOI:** 10.1155/2023/4474255

**Published:** 2023-04-17

**Authors:** Guy Cohen, Jeannette Jakus, Sumer Baroud, Raanan Gvirtz, Sharon Rozenblat

**Affiliations:** ^1^The Skin Research Institute, The Dead Sea and Arava Science Center, Masada 86910, Israel; ^2^Ben Gurion University of the Negev, Eilat Campus, Eilat 8855630, Israel; ^3^SUNY Downstate Health Sciences University, Brooklyn, NY, USA; ^4^Sharon Rozenblat R&D Consultation, Tel Aviv, Israel

## Abstract

Acne vulgaris, the most common form of acne, is characterized by a mixed eruption of inflammatory and noninflammatory skin lesions primarily affecting the face, upper arms, and trunk. The pathogenesis of acne is multifactorial and includes abnormal keratinization and plugging of the hair follicles, increased sebum production, proliferation and activation of *Cutibacterium acnes* (*C. acnes*; formerly *Propionibacterium acnes*, *P. acnes*), and finally inflammation. Recent studies have found that cannabidiol (CBD) may be beneficial in the treatment of acne. The aim of this study was to explore natural plant extracts that, when combined with CBD, act synergistically to treat acne by targeting different pathogenic factors while minimizing side effects. The first stage of the study investigated the capacity of different plant extracts and plant extract combinations to reduce *C. acnes* growth and decrease IL-1*β* and TNF*α* secretion from U937 cells. The results found that *Centella asiatica* triterpene (CAT) extract as well as silymarin (from *Silybum marianum* fruit extract) had significantly superior anti-inflammatory activity when combined with CBD compared to either ingredient alone. In addition, the CAT extract helped potentiate CBD-induced* C. acnes* growth inhibition. The three ingredients were integrated into a topical formulation and evaluated in *ex vivo* human skin organ cultures. The formulation was found to be safe and effective, reducing both IL-6 and IL-8 hypersecretion without hampering epidermal viability. Finally, a preliminary clinical study of this formulation conducted on 30 human subjects showed a statistically significant reduction in acne lesions (mainly inflammatory lesions) and porphyrin levels, thereby establishing a tight correlation between *in vitro*, *ex vivo*, and clinical results. Further studies must be conducted to verify the results, including placebo-controlled clinical assessment, to exclude any action of the formulation itself.

## 1. Introduction

Acne vulgaris, one of the most common human skin diseases, reduces the quality of life of millions of people worldwide. It affects almost everyone between the ages of 15 and 17, with 15–20% of affected individuals experiencing moderate to severe disease [1]. Although acne is principally a disorder of adolescence, current research indicates that the prevalence of acne in adult patients, especially women, is increasing. The clinical manifestations of acne include noninflammatory lesions (open and closed comedones) and inflammatory lesions (papules and pustules) [2]. Major factors in the development of acne include abnormal hyperkeratosis and shedding of the follicular epithelium, increased sebum production, proliferation of the bacteria *Cutibacterium acnes* (*C. acnes;* formerly known as *Propionibacterium acnes*, *P. acnes*), and finally inflammation. Current guidelines recommend a combination of ingredients and treatments aimed at addressing these different pathogenic factors through unique mechanisms of actions. While many options exist, most available acne treatments cause some degree of irritation, leading to low compliance rates [3]. Over the last decade, a rising interest in natural and plant-derived ingredients has led to the discovery and development of new products that provide good efficacy with less irritation, resulting in better compliance and outcomes [4–6].

Due to the important role of the endocannabinoid system in the skin, current research has focused on the role of cannabinoids in the treatment of several skin disorders [7]. Recently, cannabidiol (CBD) has been suggested as a new treatment option for acne [8]. In addition to its known anti-inflammatory activity, Oláh et al. have shown that CBD can reduce lipolysis and sebocyte proliferation *in vitro* [9]. Several clinical studies are now underway to evaluate the therapeutic potential of these properties [10].

The development of cannabinoids as therapeutic agents in dermatology and their use in cosmetic applications have been challenged by limited efficacy data and unclear regulatory and legal guidelines surrounding their use [11]. To circumvent these challenges, our team explored the use of established active molecules to provide synergistic properties, both in efficacy and tolerability, when combined with CBD in the treatment of acne. In this study, the compatibility of these herbal extracts with CBD was evaluated *in vitro*, *ex vivo*, and clinically. A statistically significant correlation of these findings using our final mixture was demonstrated across all stages of development.

## 2. Materials and Methods

All cell and skin culture mediums and supplements were supplied by Biological Industries (Beit HaEmek Israel). Unless mentioned, all chemical reagents came from Sigma-Aldrich (Israel). *Centella asiatica* triterpenes (leaf extract, containing 36–44% asiaticoside and 56–64% asiatic acid and madecassic acid) and silymarin from *Silybum marianum* fruit extract (50–60% silymarin calculated as silibinin: 20–45% silicristin, 40–65% silibinin A and B, and 10–20% isosilibinin A and B) were produced and supplied by Indena Group. *Lonicera japonica* (honeysuckle) flower extract, *Salvia miltiorrhiza* root extract (1% danshensu), and *Camellia sinensis* (green tea) extract (50% caffeine) came from Draco Natural Products. *Salix babylonica* (white willow) bark extract (25% salicin) was supplied from Botaniex, while hemp oil and CBD came from Echo Pharmaceuticals. U937 (a promonocytic, human myeloid leukemia cell line), as well as *C. acnes*, was supplied by ATCC.

### 2.1. Cell, Bacteria, and Skin Organ Cultures

U937 cells were grown and maintained in RPMI-1640 supplemented with 10% fetal calf serum and 1% streptomycin/penicillin. The cells were maintained at 5% CO_2_ in a humidified incubator at 37°C. For passaging, the cells were diluted in fresh media at a ratio of 1 : 4. Prior to the experiment, the cells were seeded at 300,000 cell/ml (150 *μ*l pr 96-well plates) and differentiated by PMA (phorbol 12-myristate 13-acetate) into a macrophage-like phenotype, an observation similarly reported in other studies [12]. After 24 hours, the adhered cells were treated as indicated. *C. acnes* was grown in a modified and reinforced clostridial broth medium with reduced levels of oxygen.

Human skin samples were obtained from 40- to 60-year-old healthy women undergoing aesthetic abdominal surgery, after signing an informed consent form. The experiments were conducted with the approval of the IRB Committee of the Soroka Medical Center, Beer Sheva, Israel (#0258-19-SOR, approval protocol scrc20016; 29.06.2020). The tissue was processed and maintained in an air/liquid interface with the dermal side submerged, as previously detailed [13, 14].

### 2.2. Viability Determination

U937 cells and skin tissue epidermal viability were monitored by the MTT assay. Following treatment, the cells were washed, and the cellular indicator was added (150 *μ*l of 0.5 mg/mL (3-(4,5- dimethylthiazol-2-yl)-2,5-diphenyltetrazolium bromide dissolved in PBS) to the 96-well plates. The plates were then incubated for 1 hour at 37°C, after which the solution was aspirated. One hundred and fifty microliters of isopropanol was then added to the solution to extract the formazan dye. The absorbance was recorded at 570 nm using a plate reader (TECAN, f200 Infinite, Switzerland). Epidermal viability was determined similarly following heat separation of the epidermis (1 min., 56°C) and its transfer to the 96-well plates.

### 2.3. Inflammation Induction and Cytokine Quantification

The cells were seeded at 300,000 cell/ml (150 *μ*l pr 96-well plates) and differentiated by PMA (phorbol 12-myristate 13-acetate, 10 ng/ml) into a macrophage-like phenotype. Then, the cells were treated without or with 1 *μ*g/mL in the absence or presence of the different treatments listed below. In addition, dexamethasone (10 *μ*M) was used as a positive control. After different treatments, the spent medium from the cell and tissue cultures was centrifuged (to remove particles) and the supernatant (100 *μ*l) was aliquoted and stored at −20°C until used. Human ELISA tests were performed according to the manufacturer's instructions (Biolegend, San Diego, CA).

### 2.4. MIC (Minimum Inhibitory Concentration) Assay

To evaluate the impact of the different treatments on *C. acnes*, a stock of frozen bacteria was removed from a −80°C freezer and thawed into a *U*-shaped falcon tube containing 4 ml of the modified, reinforced clostridial broth medium at 37°C under reduced oxygen levels *(anaerobic chamber)*. After 3 hr, the bacteria were inoculated into agar plates (100 *μ*l/10 cm plate) and grown under reduced oxygen levels. A single colony was grown in 4 ml media to a midlog phase (O.D. 0.5; 600 nm, approx. 3 hr). Then, the bacteria were diluted to O.D. 0.05 in the absence or presence of different treatments. Bacterial growth, determined by absorbance (O.D. 600), was monitored kinetically using a microplate reader (Infinite f200, TECAN), heated to 37°C. Ampicillin, at 10 *μ*g/ml, was used as a positive control.

### 2.5. Formulation

A topical formulation containing 1% CBD, 1% CAT, and 1% silymarin was prepared by Echo Pharmaceuticals. The commercial formulation was developed as a hydroalcoholic gel using xanthan gum and acrylates/sodium acryloyldimethyl laurate copolymers as rheology modifiers. The formulation also has contained 0.5% of salicylic acid. The active ingredients were added in a step-wise manner at 45°C under constant stirring to increase solubility. A step-by-step preparation can be obtained by the authors, as well as samples of the formula for academic usage, after standardized NDA.

### 2.6. Clinical Study

The study was performed according to the Declaration of Helsinki principles and subsequent amendments. The study was conducted in the spirit of Good Clinical Practice Guidelines and general principles of Law 46/2004 of August 19^th^. The protocol and test conditions were reviewed by the Internal Review Board (opinion no. 6776/2021), and the standard protocol was submitted to the ethical commission of PhD trials (from December 27^th^, 2019). This study was conducted in accordance with the general conditions of PhD trials and was established as a research project involving human subjects, summarised by the protocol (MD.122/02 Protocol PT.06.01/final04).

#### 2.6.1. Inclusion Criteria

Eligible study participants included male or female subjects aged 15–40 years with mild to moderate facial acne (Grade 2 or 3 according to the Investigator's Global Assessment (IGA)) and skin Fitzpatrick phototype II to IV. All enrolled participants and/or their parent/legal guardian (for minor subjects) provided written, informed consent and willingly agreed to comply with study requirements.

#### 2.6.2. Exclusion Criteria

Subjects were excluded from the study if 1 or more of the following treatments were used: oral retinoids (within 6 months prior), topical retinoid treatment or any facial aesthetic or medical treatments (within 1 month prior), and excessive UV exposure (within 1 month prior). Pregnant women, breastfeeding women, or women planning a pregnancy during the clinical study were also excluded. Participants with medical conditions or those receiving treatments that, according to the investigator's judgment, could compromise the safety of the participant or interfere with outcomes of the study were also excluded. Furthermore, subjects with a planned or expected major surgical procedure during the clinical study were not included.

#### 2.6.3. Study Population

It is presented in Table 1. Thirty-three subjects aged 15–40 with mild to moderate acne were enrolled in the study, with three withdrawals (#5 on D0, #19 on D28 and #11 on D56). Data from the remaining 30 subjects were included in our analysis.

#### 2.6.4. Methods

Study participants were instructed to apply a thin layer of the study cream (ECHO-A-01) to any spot (red or not) and any imperfection on the skin 2 or 3 times a day (morning, noon, and evening (before bedtime)) on clean skin. Instrumental efficacy data were collected on days D0, D28, D42, and D56 and analyzed using the Wilcoxon signed-rank test. All the calculations were performed using SPSS 23 (IBM) with a 95% confidence interval. High-resolution photographs were taken at each visit using the VISIA-CR imaging system, a standardized clinical imaging and image analysis system that uses a standard light (IntelliFlash), a cross-polarized flash, and a parallel, polarized flash under ultraviolet lighting. The software allows a region of interest to be defined and calculated using numbered parameters and quantify porphyrin levels.

## 3. Results and Discussion

### 3.1. *In Vitro* Screening for CBD-Based Herbal Composition

**Figure 1:**
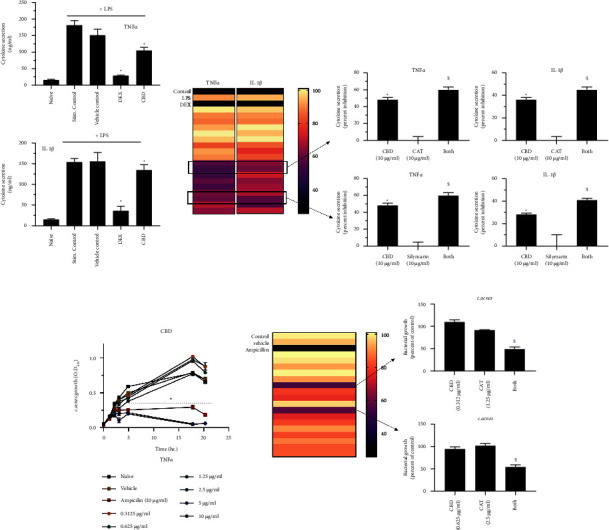
*In vitro* screening of CBD-based natural mixtures with herbal extracts. (a) U937 cells (300,000 cell/ml) were stimulated with 1 *μ*g/mL LPS and treated for 24 hr. Cytokine levels were evaluated by ELISA. Dexamethasone (DEX) was used as a positive control. (b) The natural mixtures were evaluated similarly, and the compatible blends are presented as a heat map (dark colors depict high inhibition) and selected effective mixture on the right panel. (c) The impact of increasing concentrations of CBD on *C. acnes* growth was evaluated kinetically by the MIC assay. (d) Bacterial growth after 6 hr was similarly evaluated in the presence of the natural mixtures. *n* = 3; ^*∗*^/$ depicts significance in comparison to the control or CBD group, respectively.

Two initial *in vitro* screening models were used to evaluate the compatibility and possible synergistic action of herbal mixtures: LPS-induced inflammation of U937 cells and the growth inhibition of *C. acnes.* First, the impact of CBD (10 *μ*g/ml) alone was evaluated in these systems and is demonstrated in Figures 1(a)–1(c). Exposure to CBD significantly decreased the secretion of both TNF*α* and IL-1*β* (Figure 1(a)) in the *in vitro* LPS-induced inflammatory model. Concentrations of 5 and 10 *μ*g/ml of CBD also reduced *C. acnes* growth in a comparable manner to ampicillin, which was used as a positive control (Figure 1(c)). These results complement previous findings and reports, suggesting CBD as a potential treatment for acne. Olah et al. demonstrated that CBD inhibits lipogenesis in sebocytes stimulated with either arachidonic acid or a combination of linoleic acid and testosterone. In addition, CBD reduced cell proliferation via the activation of transient receptor potential vanilloid 4 channels [9]. A recent review also shed light on other inflammatory disorders that can be mitigated by CBD-based treatment, including allergic contact dermatitis, psoriasis, acne, scleroderma, and dermatomyositis [15]. In addition, a study dating back to 1976 found that CBD may display antibacterial properties [16], and a more recent, comprehensive study by Blaskovich et al. found that CBD can reduce growth of several bacteria, including highly resistant *Staphylococcus aureus*, *Streptococcus pneumoniae*, and *Clostridioides difficile*. The authors also concluded that membrane impairment is the primary bactericidal mechanism of CBD [17].

Our studies confirm both the anti-inflammatory and antibacterial properties of CBD, thereby supporting its use in the treatment of acne. However, as acne is a multifactorial skin condition, we sought to create a more comprehensive treatment formulation by adding other already existing chemicals with unique mechanisms of action to act synergistically with CBD to enhance its activity but at a lower dose in order to reduce local side effects and improve tolerability. Therefore, the efficacy of subeffective concentrations of CBD when used alone or combined with seven commercially available herbal extracts was investigated (preliminary screen, data not shown). As shown in Figure 1(b), only two extracts were found to be highly compatible with CBD, the *Centella asiatica* triterpene (CAT) extract and silymarin extracted from *Silybum marianum* fruit. The combination of either extract with CBD potentiated its effect in reducing LPS-induced inflammation. Moreover, the CAT extract enhanced the growth arrest of *C. acnes* when combined with CBD (Figure 1(d)). The CAT extract is well known for its therapeutic effect mainly due to the presence of madecassoside, asiaticoside, madecassic acid, and asiatic acid [18–21]. Our study shows that CAT potentiates the mitigating effect of subtherapeutic concentrations of CBD. Since the combination was active in two separate models, it is possible that it increases the bioavailability of CBD. Further studies are required to understand the mechanism of action underlining this phenomenon. The *Silybum marianum* fruit extract had been repeatedly shown to modulate the inflammatory system [20, 21] and therefore can be beneficial in the treatment of several skin disorders, including the inflammatory lesions of acne vulgaris. Our study identified a synergistic effect between CBD and silymarin on two cytokines (Figure 1(b)).

### 3.2. *Ex Vivo* Validation of the Compounds

**Figure 2:**
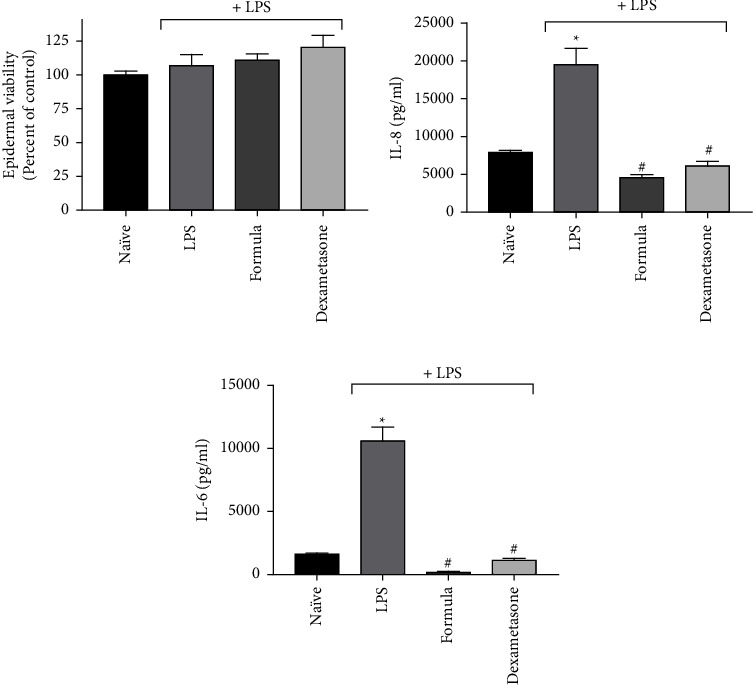
The topical formulation containing the natural mixture (“formula”) attenuates LPS-induced inflammation. Inflammation was inflicted in the *ex vivo* human skin model by 5 *µ*g/ml LPS and treated with the formulated natural mixture for 48 hr. (a) Epidermal viability was evaluated by MTT. (b, c) The quantification of IL-8 and IL-6 by ELISA, respectively. *n* = 3; ^*∗*^/# depicts significance in comparison to the control or LPS-stimulated group, respectively.

A topical formulation containing 1% CBD, 1% CAT, and 1% silymarin was prepared and evaluated in the *ex vivo* human skin organ culture (hSOC). The final formulation contained additional 0.5% salicylic acid, which was foreseen to facilitate the therapeutic action of the mixture through several mechanisms [22, 23]. The hSOC system was used repeatedly for both efficacy and safety evaluation, as it emulated the intact tissue [24–26]. The results presented in Figure 2 show that the formulation containing the natural mixture (written as “formula” in Figure 2) was well tolerated by the skin and did not reduce epidermal viability (determined by the MTT method). Although LPS stimulation increased the secretion of IL-6 and IL-8, the cytokines were subsequently blocked by the formula, an effect comparable to that of dexamethasone. Both IL-6 and IL-8 are the main cytokines known to be induced by LPS in the *ex vivo* system [13]. Importantly, both cytokines have been suggested as possible therapeutic targets to acne vulgaris [27–29]. Therefore, their attenuation, as exhibited in our study, may make our formula clinically relevant as a potent anti-inflammatory formulation.

### 3.3. Clinical Evaluation of the Formula

Afterwards, the formula was clinically evaluated on 30 subjects, both male and female aged 15–40 years, with mild to moderate acne (Grade 2 or 3 according to the Investigator's Global Assessment (IGA)). It was applied 2 to 3 times a day directly on spots and any imperfection on the skin (as a spot treatment) for 56 days.

**Figure 3:**
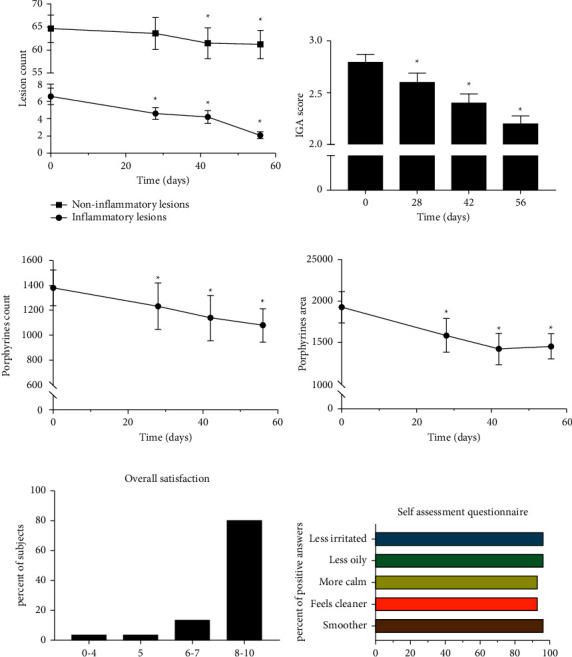
The topical formulation of the natural mixture attenuates acne lesions. The summary of a 56-day clinical evaluation is presented. (a) Changes in noninflammatory and inflammatory lesions are depicted. (b) The Investigator's Global Assessment (IGA) is presented. (c, d) Porphyrin abundance is depicted. (e, f) The summary of the volunteer questionnaire is shown.

**Figure 4:**
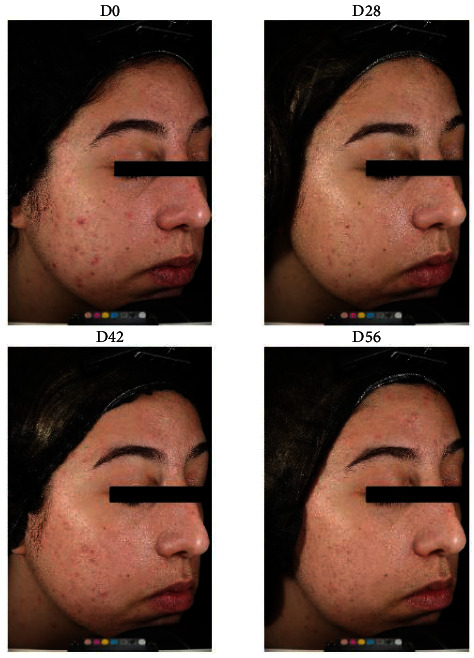
Representative image of clinical evaluation of the natural mixture.

The formula showed a statistically significant reduction in acne lesions, mainly inflammatory. A reduction of 31.8% in total inflammatory lesions was shown following 28 days, 38.2% following 42 days, and 70.9% following 56 days (Figure 3), suggesting that the formula's potent anti-inflammatory activity has significant therapeutic potential in inflammatory acne. The topical application significantly reduced acne severity, shown as a reduction in the Investigator's Global Assessment (IGA) score. In addition, a marked reduction in porphyrin levels (count and area) suggested *C. acnes* inhibition. Representative images are presented in Figure 4. It should also be noted that the overall rate of volunteer satisfaction was extremely high throughout the trial.

## 4. Conclusions

The data presented here demonstrate the efficacy of a newly developed, natural topical formulation in the treatment of acne. Since several pathological pathways underlie the physiology of acne, a treatment that targets several mechanisms of action is advantageous. The topical formulation presented in this study showed significant ability in reducing inflammation and eliminating *C. acnes*. The *ex vivo* results show a marked impact of the developed formulation in comparison to untreated tissue. However, the addition of salicylic acid may have further enhanced the efficacy of the formulation, due to its keratolytic activity, and as a result, it may have exhibited positive outcomes in the treatment of acne. Thus, a major limitation of this study is the lack of formulation without CBD and *Silybum marianum* fruit extract to exclude indirect effects of other compounds. Thus, placebo-controlled evaluation must be performed to validate the study. A significant correlation was shown between *in vitro*, *ex vivo*, and clinical tests, where the inhibition of inflammation and *C. acnes* growth were the main mechanisms of action exhibited by our formulation. The study also demonstrates a transformation factor of 1 : 1000 (∼10 *μ*g/ml to 1%) when converting from *in vitro* concentrations to *ex vivo* and clinical ones. The selected concentration in the formulation was determined after a preliminary evaluation determining that the formulation was safe and well tolerated by the tissue on one hand and effective on the other one (data not shown). This finding is also supported by previous findings, transforming data obtained in HaCaT keratinocyte cells to *ex vivo* experiments [28]. The study has several limitations: *in vitro* screening was aimed to find CBD-compatible agents, and thus, low levels of the herbal extracts were used. In addition, the significant results obtained clinically were compared to their basal state, as typically performed in cosmetic trials, with no placebo control used. However, the massive reduction in inflammatory acne lesions, as well as porphyrin levels, clearly stands out and was foreseen based on the dual *in vitro* and *ex vivo* data.

## Figures and Tables

**Table 1:** Study population, including demographic and skin reactivity.

Demographic data (subjects who applied the product at least once)	Skin	Subject having mild to moderate facial acne (Grade 2 or 3 according to the Investigator's Global Assessment (IGA))	Subjects
Number 33 (100%)	Skin reactivity:	33 (100%)	Included 33 (100%)
Females 33 (81.8%)	Reactive 6 (18.2%)		Analyzed 30 (90.9%)
Males 6 (18.2%)	Sensitive 8 (24.2)		Dropped 3 (9.1%)
Adults 25 (75.8%)	Normal 19 (57.6%)		
Adolescents 8 (24.2%)			
Mean age 22.8	Skin condition:		
Age min 15	Combined 25 (75.8%)		
Age max 40	Oily 8 (24.2%)		
Phototype II 8 (24.2%)			
Phototype III 21 (63.6%)			
Phototype IV 4 (12.1%)			

## Data Availability

All clinical data are available upon request.
